# *Theileria orientalis* Ikeda in Cattle, Alabama, USA

**DOI:** 10.3390/vetsci10110638

**Published:** 2023-10-30

**Authors:** Nneka Iduu, Subarna Barua, Shollie Falkenberg, Chance Armstrong, Jenna Workman Stockler, Annie Moye, Paul H. Walz, Chengming Wang

**Affiliations:** 1 Department of Pathobiology, College of Veterinary Medicine, Auburn University, Auburn, AL 36849, USA; nvi0001@auburn.edu (N.I.); szb0116@auburn.edu (S.B.); smf0076@auburn.edu (S.F.); walzpau@auburn.edu (P.H.W.); 2 Department of Clinical Sciences, College of Veterinary Medicine, Auburn University, Auburn, AL 36849, USA; armstcl@auburn.edu (C.A.); jew0027@auburn.edu (J.W.S.); 3 College of Sciences and Mathematics, Auburn University, Auburn, AL, USA; acm0146@auburn.edu

**Keywords:** *Theileria orientalis* Ikeda, cattle, Alabama, *Haemaphysalis longicornis* tick

## Abstract

**Simple Summary:**

*Theileria orientalis* Ikeda poses significant challenges to the cattle industry, encompassing economic repercussions, trade-related issues, and constraints regarding control and treatment measures. This parasite can precipitate a range of adverse effects in cattle, including diminished milk production, weight loss, anemia, and, in severe instances, cattle fatalities. These adverse impacts directly translate into economic burdens for farmers and the broader cattle industry. The financial costs associated with treating afflicted animals and implementing control strategies can be substantial. Additionally, occurrences of *Theileria orientalis* Ikeda in regions where it is not endemic can lead to trade restrictions and impediments affecting both cattle and cattle products. This can curtail the international movement of cattle and influence global trade in livestock. Furthermore, there is a dearth of effective treatment options for infected cattle, and severe cases frequently culminate in fatalities. This lack of viable treatment exacerbates the difficulties in disease management. Addressing the presence of *Theileria orientalis* Ikeda in the USA necessitates comprehensive efforts to understand the prevalence and distribution of the pathogen. This study utilizing PCR followed by DNA sequencing identified two cases of *Theileria orientalis* Ikeda-positive cattle, marking the first report of this virulent genotype in Alabama, USA.

**Abstract:**

*Theileria orientalis* Ikeda genotype, a parasite causing a disease in cattle that leads to significant economic challenges in Asia, New Zealand, and Australia, has been identified in seven U.S. States since 2017. Two previously validated PCR tests for *Theileria* followed by DNA sequencing were performed to test blood samples collected from 219 cattle in Alabama, USA, during the period of 2022–2023. Bidirectional Sanger sequencing revealed that the MPSP gene sequences (639–660 bp) from two cattle in Lee and Mobile Counties of Alabama exhibited a 100% match with those of recognized *T. orientalis* Ikeda strains, and showed similarities ranging from 76% to 88% with ten other *T. orientalis* genotypes. A high copy number of *T. orientalis* Ikeda was detected in the blood of infected cattle (ALP-1: 1.7 × 10^5^ and 1.3 × 10^6^/mL whole blood, six months apart; ALP-2: 7.1 × 10^6^/mL whole blood). Although the confirmed competent vector for *T. orientalis* Ikeda, *Haemaphysalis longicornis* tick, has not yet been identified in Alabama, the persistent nature of *T. orientalis* Ikeda infection and the detection of a high pathogen burden in seemingly healthy cattle in this study suggest that other tick species, as well as shared needles and dehorning procedures, could facilitate pathogen transmission within the herd. Continued investigations are necessary for the surveillance of *T. orientalis* Ikeda and *Haemaphysalis longicornis* ticks in Alabama and other U.S. states, along with assessing the pathogenicity of *T. orientalis* Ikeda infections in cattle.

## 1. Background

*Theileria orientalis* is a hemoprotozoan parasite belonging to the phylum Apicomplexa [1]. The infection of cattle with this parasite can lead to various adverse effects, including anemia, weakness, reduced milk production, diminished weight gain, abortion, and occasionally, death [2,3,4,5,6]. Researchers have identified eleven genotypes of *T. orientalis* (Sivakumar et al., 2014), with the most frequently encountered genotypes being Buffeli, Chitose, and Ikeda [1,6,7,8]. While *T. orientalis* Buffeli is typically clinically benign, Chitose and Ikeda tend to be more virulent, resulting in increased morbidity and mortality in affected cattle [2,8].

Although sporadic and limited, outbreaks of *T. orientalis* Buffeli were reported in the United States (U.S.) before 2017 [9,10,11]. Recently, *T. orientalis* Ikeda has become endemic in Virginia [5,6,11,12], and has also been detected in seven U.S. states to date [12].

To assess the *T. orientalis* status in cattle, sheep, and goats in Alabama, USA, we conducted PCR assays followed by DNA sequencing analysis on existing convenience samples of whole blood and blood samples obtained from farms in Alabama.

## 2. Materials and Methods

### 2.1. Whole Blood Samples

Samples for analysis came from two sources. Whole-blood samples in EDTA from cattle (n = 72) presenting to the Auburn University Large Animal Teaching hospital between September 2022 and August 2023 for regular physical examination and the diagnosis and treatment of diseases where whole blood was submitted to the Clinical Pathology Laboratory were used in the study. Aliquots of the whole-blood samples were used for routine complete blood counts and biochemical profiles. Additionally, 147 bovine blood samples from three cattle farms associated with Auburn University College of Veterinary Medicine located in Marion County (n = 50), Lee County (n = 33), and Tallapoosa County (n = 64) of Alabama were also used to screen *Theileria* in this study. The protocol for the collection of bovine samples was reviewed and approved by the Institutional Animal Care and Use Committee of Auburn University (IACUC #2022-5112).

Blood samples were sent to Auburn at ambient temperature. The High-Pure PCR Template Preparation Kit (Roche Molecular Biochemicals, Indianapolis, IN, USA) was used to extract DNA from 400 µL aliquots as published [13,14]. The extracted DNA was eluted in 200 µL elution buffer, and was preserved at −80 °C until PCR was performed in this study.

### 2.2. Detection of Theileria and Anaplasma DNA by PCRs

Two previously validated quantitative PCRs, an MPSP gene-based *Theileria* TaqMan PCR [15] and a FRET-PCR targeting the rRNA of *Theileria* [14], were used to detect *Theileria* DNA in this study. The primers and probes as well as the primers to sequence the MPSP gene are shown in Table 1.

All PCR reactions were performed on a Roche Light Cycler 480 II Thermocycler as described [15]. In brief, 10 µL of the extracted DNA was added to a 10 µL reaction mixture containing 5× PCR FRET buffer, 400 µM dNTP (Roche Diagnostics GmbH, Mannheim, Germany), 0.34 units of Platinum *Taq* DNA Polymerase (Invitrogen, Boston, MA, USA), 1 µM of each forward and reverse primer (Integrated DNA Technologies, Coralville, IA, USA). Thermal cycling consisted of 18 high-stringency step-down cycles followed by 25 relaxed-stringency fluorescence acquisition cycles. The 18 high-stringency step-down thermal cycles were 6 × 15 s @ 95 °C, 60 s @ 75 °C; 9 × 15 s @ 95 °C, 60 s @ 73 °C; and 3 × 15 s @ 95 °C, 30 s @ 71 °C, 30 s @ 72 °C; this was followed by the relaxed-stringency fluorescence acquisition cycling of 25 × 15 s @ 95 °C, 8 s @ 58 °C; this is was followed by fluorescence acquisition, 30 s @ 65 °C, and 30 s @ 72 °C.

**Figure 1:**
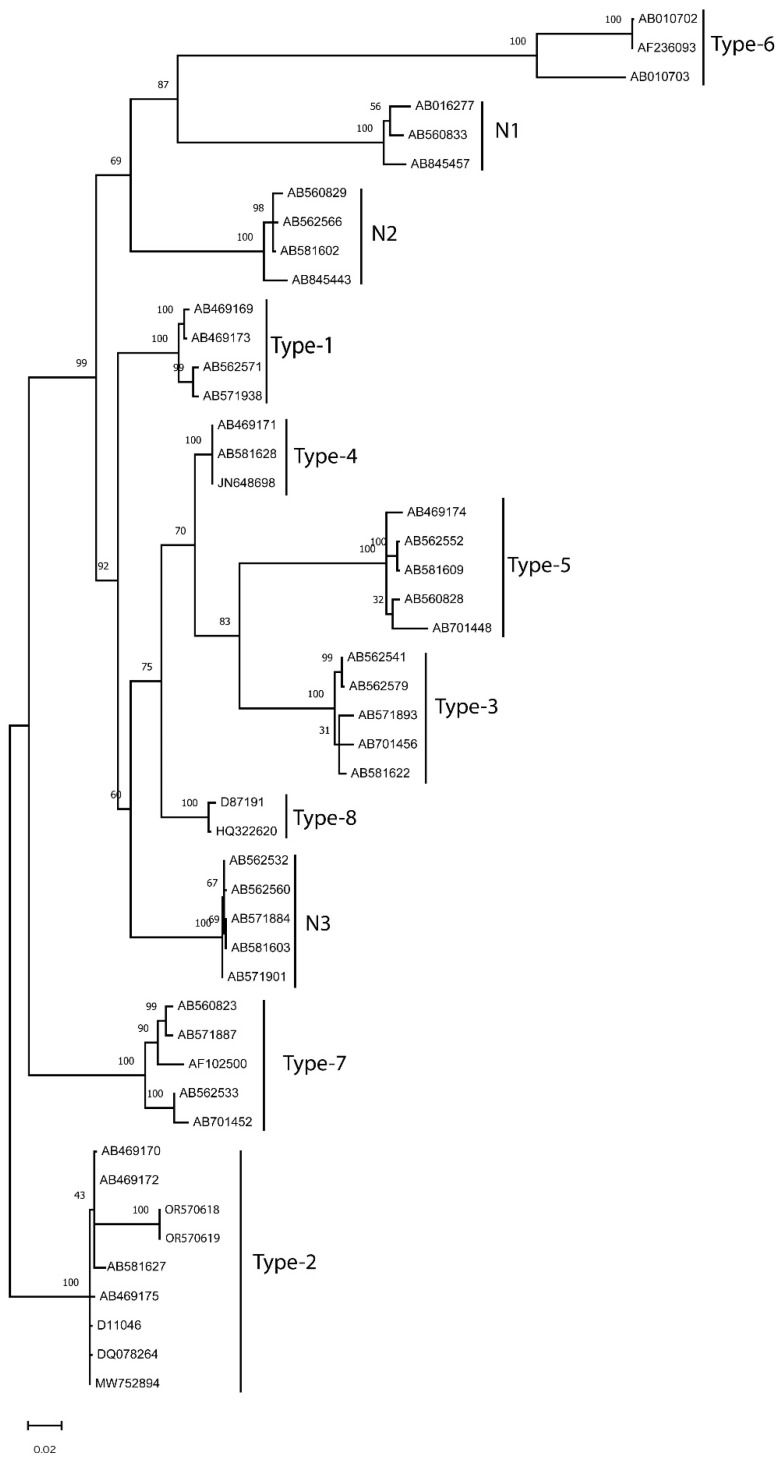
Phylogenetic tree displaying major piroplasm surface protein (MPSP) gene sequences for *Theileria orientalis.* The 739–760-bp nucleotide sequences of the MPSP gene, representing 11 genotypes of *T. orientalis*, were concatenated and aligned using CLUSTALW. A maximum likelihood phylogenetic tree was generated employing a p-distance model. Bootstrap values are expressed as percentages based on 1000 replications, and the bar represents evolutionary distances as 0.05 changes per nucleotide position. The genotypes of *T. orientalis*, along with their GenBank accession numbers, are provided. Notably, the sequences of *T. orientalis* Ikeda identified in this study (ALP-1: OR570618; ALP-2: OR570619) exhibited 100% identity with those of recognized *T. orientalis* Ikeda strains, while demonstrating 76–88% similarity with other *T. orientalis* genotypes.

The products of *Theileria*-positive PCRs were sent to ELIM Biopharmaceuticals (Hayward, CA, USA) for Bidirectional Sanger sequencing. The nucleotide sequences were submitted to NCBI to obtain GenBank Accession numbers (OR570618, OR570619), and a phylogenetic tree was generated to compare the nucleotide sequences of *Theileria* identified in this study with those of all recognized 11 *T. orientalis* genotypes (Figure 1).

The *Theileria*-positive samples identified in this study were submitted to the Molecular diagnostic laboratory at Auburn University College of Veterinary Medicine for the detection of *Anaplasma* DNA following the published protocol [16].

## 3. Results

Both the MPSP-based TaqMan PCR and rRNA-targeting FRET-PCR, followed by DNA sequencing, successfully identified *T. orientalis* DNA in 2 out of 89 convenience blood samples. However, none of the 147 bovine blood samples from three different farms tested positive for *T. orientalis*. Notably, the two *T. orientalis*-positive samples were found to be free of *Anaplasma* spp.

The MPSP serves as an antigenic marker for *Theileria* spp. and is widely employed for genotyping the 11 distinct *T. orientalis* groups [1,17]. The 739–760 bp nucleotide sequences of the MPSP gene, representing these 11 *T. orientalis* genotypes, were concatenated and aligned using CLUSTALW. Subsequently, a maximum likelihood phylogenetic tree was constructed. The sequences from *T. orientalis* identified in this study (ALP-1, ALP-2) displayed a 100% match with those from recognized *T. orientalis* Ikeda strains (Figure 1). Furthermore, they exhibited similarities ranging from 76% to 88% with other *T. orientalis* genotypes (Type 1: 86–87%; Type-3: 82–83%; Type-4: 87%; Type-5: 80–81%; Type-6: 76%; Type 7: 88%; Type-8: 86%; N1: 79%; N2: 83–84%; N3: 86–87%) (Figure 1). Although the internal segment of the small subunit gene was not amplified in the *Theileria*-positive samples in this study, the phylogenetic trees, based on the long MPSP fragment, unequivocally confirmed the presence of *T. orientalis* Ikeda DNA in two cattle from Alabama.

In Lee County, Alabama, a one-year-old female heifer (ALP-1 with an accession number OR570618) was found to carry *T. orientalis* Ikeda DNA. This heifer exhibited normal red blood cell (RBC) and hemoglobin (HGB) counts but displayed elevated monocyte counts (2.3/µL) and white blood cell counts (2.1 × 10^3^/µL). Additionally, increased levels of glucose (313 mg/dL) were observed, alongside low iron levels (25 µg/dL). This heifer was being treated for an acute free gas bloat resulting from grain overload. She recovered from this episode of ruminal acidosis following treatment and has been a healthy member of the resident herd since discharge. The initial test revealed the presence of 1.7 × 10^5^ *T. orientalis* Ikeda/mL in whole blood, and a follow-up test six months later showed the increased count of 1.3 × 10^6^/mL in whole blood.

A four-month-old male calf from Mobile County, Alabama, presented with fever, swollen lymph nodes, anorexia, pyrexia, cough, dyspnea, lethargy, tachypnea, polyuria, and diarrhea. Bloodwork, including chemistry and a complete blood count, revealed no significant abnormalities except for decreased fibrinogen levels, indicating recovery from a previous inflammatory event. A urinalysis was also performed, revealing dilute urine but no signs of infection or inflammation of the bladder. This calf tested positive for *T. orientalis* Ikeda (ALP-2 with an accession number OR570619), with a copy number of 7.1 × 10^6^/mL in whole blood.

## 4. Discussion

*T. orientalis* Ikeda is a protozoan parasite of cattle that has significant implications for the livestock industry. In this study, we reported for the first time the identification of this virulent genotype Ikeda in the cattle from Alabama, USA. Persistent infection with different variants of *T. orientalis* is a common occurrence [7,18], and the immune mechanisms responsible for disease resistance are not yet fully understood. In this study, a follow-up test conducted six months later revealed an increased count of 1.3 × 10^6^/mL of *T. orientalis* Ikeda in the whole blood of one animal, compared to the initial count of 1.7 × 10^5^ *T. orientalis* Ikeda/mL whole blood, with both cattle appearing to be in relatively good health, and no evidence of anemia or fever being observed. This supports the notion that *T. orientalis* can lead to chronic and persistent infections, emphasizing the need for further research into the pathogenicity of *T. orientalis* Ikeda infections in ruminants.

An experimental transmission trial conducted by the USDA’s Agricultural Research Service (ARS) in collaboration with the Virginia Tech Animal Laboratory Services (ViTALS) laboratory has confirmed the vector competence of the *Haemaphysalis longicornis* tick (ALHT: Asian long-horn tick) for *T. orientalis* Ikeda [17]. ALHT can feed on a wide range of wildlife and domestic species, including birds, white-tailed deer, companion animals, livestock, equines, and humans. As of September 2023, ALHT has been identified in 18 states [19]. Once introduced into suitable habitats, ALHT populations can proliferate rapidly, partly due to the exhibition of multiple genetic types, including both parthenogenetic and bisexual reproduction [20,21].

Although ALHT has not yet been identified in Alabama, given the persistent nature of *T. orientalis* Ikeda infection and the detection of a high burden of *T. orientalis* Ikeda in apparently healthy cattle in this study, it is plausible that other tick species, as well as shared needles and dehorning procedures, could facilitate the spread of the pathogen within the herd. Telionis et al. reported an 8.7% (172/1980) prevalence of genotype Ikeda in Virginia Market Cattle, 2018–2020 [6]. It is likely that *T. orientalis* Ikeda will be identified in many other states and become established throughout the USA.

## 5. Conclusions

In conclusion, PCR tests followed by DNA sequencing confirmed the presence of *T. orientalis* Ikeda DNA in the blood of two cattle in Alabama, USA. Continued research in epidemiology, vector ecology and vaccine development will contribute to a better understanding of *Theileria orientalis* Ikeda and help develop more effective strategies for its control and prevention, benefiting both the cattle industry and animal health.

## Figures and Tables

**Table 1:** Primers and probes used in this study.

PCR and the Target Gene	Oligonucleotides	References
TaqMan-PCR; MPSP gene of all *T. orientalis* genotypes	MPSP-F: 5′-GCAAACAAGGATTTGCACGC-3′	Bogema et al., 2015 [15]
	MPSP-R: 5′-TGTGAGACTCAATGCGCCTAGA	
	Pr-U: 5′-FAM-TCGACAAGTTCTCACCAC-MGB-NFQ-3′	
FRET-PCR; rRNA for all *Theileria* spp.	F: 5′-TAGTGACAAGAAATAACAATACGGGGCTT-3′	Yang et al., 2014 [14]
	R: 5′- CAGCAGAAATTCAACTACGAGCTTTTTAACT-3′	
	Pr-1: 5′-CCAATTGATACTCTGGAAGAGGTTT-(6-FAM)-3′	
	Pr-2: 5′-(LCRed640)-AATTCCCATCATTCCAATTACAAGAC-phosphate-3′	
Sequencing primers for Ikeda genotype	RTF-I: ATTGGTAGACGGAAAATGGAAGAAGG	Bogema et al., 2015 [15]
	RTR-I: GAGACTCAATGCGCCTAGAGATAATAGA	

## Data Availability

The data presented in this study are available on request from the corresponding author.
